# *In Utero* Exposure to Bisphenol A Shifts the Window of Susceptibility for Mammary Carcinogenesis in the Rat

**DOI:** 10.1289/ehp.1002148

**Published:** 2010-07-30

**Authors:** Angela M. Betancourt, Isam A. Eltoum, Renee A. Desmond, Jose Russo, Coral A. Lamartiniere

**Affiliations:** 1 Department of Pharmacology and Toxicology; 2 UAB Comprehensive Cancer Center; 3 Department of Pathology and; 4 Division of Preventive Medicine, University of Alabama at Birmingham, Birmingham, Alabama, USA; 5 Breast Cancer Research Laboratory, Fox Chase Cancer Center, Philadelphia, Pennsylvania, USA

**Keywords:** bisphenol A, cell proliferation, endocrine disruptors, mammary cancer, susceptibility

## Abstract

**Background:**

Bisphenol A (BPA) is a ubiquitous environmental chemical with reported endocrine-disrupting properties.

**Objective:**

Our goal in this study was to determine whether prenatal exposure to BPA predisposes the adult rat mammary gland to carcinogenesis.

**Methods:**

Pregnant rats were treated orally with 0, 25, or 250 μg BPA/kg body weight (BW) from gestation day (GD) 10 to GD21. For tumorigenesis experiments, prenatally exposed female offspring received a single gavage of 7,12-dimethylbenz(*a*)anthracene (DMBA; 30 mg/kg BW) on postnatal day (PND) 50, or PND100.

**Results:**

Prenatal exposure of the dam to 250 μg BPA/kg BW combined with a single exposure of female offspring to DMBA on PND100, but not on PND50, significantly increased tumor incidence while decreasing tumor latency compared with the control group. Prenatal exposure of the dam to 250 μg BPA/kg BW, in the absence of DMBA to the female offspring, increased cell proliferation and elicited differential effects at the protein level at PND100 compared with PND50. Differentially regulated proteins in the mammary gland included estrogen receptor-α, progesterone receptor-A, Bcl-2, steroid receptor coactivators, epidermal growth factor receptor, phospho-insulin-like growth factor 1 receptor, and phospho-Raf.

**Conclusions:**

Our study demonstrates that oral prenatal exposure to BPA increases mammary cancer susceptibility in offspring and shifts the window of susceptibility for DMBA-induced tumorigenesis in the rat mammary gland from PND50 to PND100. These changes are accompanied by differential effects of prenatal BPA exposure on the expression of key proteins involved in cell proliferation.

Fetuses, newborns, infants, and adolescents are populations uniquely susceptible to biochemical insult from environmental chemicals. Although some of these toxicities may be evident immediately, some exposures that take place during early critical periods of development can result in subtle alterations that are delayed in expression. Hence, at the time of clinical manifestation in adulthood, the original chemical effector may no longer be measurable.

One such environmental chemical is bisphenol A (BPA), a chemical originally developed for use as a synthetic estrogen and now one of the highest-volume chemicals produced in the world (Burridge 2003; Welshons et al. 2006). BPA is used in the manufacture of polycarbonate plastics and resins, which are commonly used in the packaging of canned foods and in beverage bottles. BPA may be ingested by humans, as it reportedly leaches from the lining of food and soda cans, (Biles et al. 1997; Brotons et al. 1994), polycarbonate bottles (Kang et al. 2006), and dental sealants (Olea et al. 1996). As a consequence of this widespread opportunity for exposure, an estimated 95% of Americans tested, including young girls, show detectable concentrations of BPA in their urine (Calafat et al. 2005; Wolff et al. 2007). Concern regarding the potential role of BPA in breast cancer is supported by animal studies showing the developing mammary gland as one of the tissues targeted by prenatal BPA exposure (Durando et al. 2007). Markey et al. (2001) showed that mice exposed *in utero* to 250 μg BPA/kg body weight (BW) via osmotic pumps had a significantly greater number of ductal and alveolar structures relative to the control group on postnatal day (PND) 180. Similarly, Murray et al. (2007) reported the development of ductal hyperplasia in CD-1 mice on PND50 and PND95 after prenatal exposure to 2.5, 25, 50, and 1,000 μg BPA/kg BW/day. In another study, Moral et al. (2008) showed that adult offspring exposed prenatally via dams treated orally with 250 μg BPA/kg BW/day had modifications of the mammary gland architecture, mainly in the number of undifferentiated epithelial structures of the tissue. A previous study from our laboratory (Jenkins et al. 2009) showed that prepubertal exposure to BPA increased 7,12-dimethylbenz(*a*)anthracene (DMBA)-induced mammary carcinogenesis in Sprague-Dawley CD rats on PND50. Therefore, our next goal was to determine if *in utero* exposure to BPA would elicit a similar response in the mammary gland after carcinogenic insult. In the initial phase of the study, we investigated the effects of prenatal BPA exposure [25 or 250 μg/kg BW/day on gestation day (GD) 10–GD21] on tumorigenesis and protein expression at PND50 in rats. For the second phase of the study, we investigated the effects of prenatal BPA exposure (250 μg/kg BW/day) on tumorigenesis and protein expression at PND100. Differential protein expression patterns induced by prenatal exposure to BPA in the mammary gland on PND50 and PND100 suggest a possible mechanism of action for the increased susceptibility of the mammary gland to chemically induced carcinogenesis on PND100.

## Materials and Methods

### Chemicals

We obtained antibodies to phospho-c-proto-oncogene serine/threonine-protein kinase (phospho-c-Raf), phospho-insulin-like growth factor 1 receptor (phospho-IGF-1R), receptor tyrosine-protein kinase erbB-2 (phospho-ErbB2), phospho-serine/threonine-protein kinase Akt (phospho-Akt), and glyceraldehyde-3-phosphate dehydrogenase (GADPH) from Cell Signaling (Danvers, MA). Steroid receptor coactivator 1 (SRC-1), SRC-2/transcriptional intermediary factor 2 (SRC-2/TIF2), and SRC-3/amplified in breast cancer-1 protein antibodies were purchased from Becton, Dickinson and Company (Franklin Lakes, NJ). Epidermal growth factor receptor (EGFR), estrogen receptor-α (ER-α), progesterone receptor (PR), and B-cell lymphoma 2 (Bcl-2) antibodies were purchased from R&D Systems (Minneapolis, MN); and extracellular signal-regulated kinase 1/2 (phospho-ERK 1/2) antibodies were from Promega (Madison, WI). All other chemicals were purchased from Sigma Chemical Co. (St. Louis, MO).

### Animals

Animal studies were conducted in accordance with the University of Alabama at Birmingham Guidelines for Animal Use and Care. Animals were treated humanely and with regard for alleviation of suffering. Adult male and female Sprague-Dawley CD rats (Charles River, Raleigh, NC) were housed in a temperature-controlled environment (22 ± 2°C) with a 12-hr dark-light cycle (lights on between 0800 and 2000 hours). The initial experiments were designed to determine the potential of prenatal exposure to BPA to alter *a*) the endocrine system during postnatal development; *b*) ER-α, SRC, and growth factor signaling protein levels in the mammary gland; and *c*) susceptibility to chemically induced mammary cancer at PND50 in the resulting offspring. For this, pregnant rats (a minimum of 30/group) were gavaged with BPA at 25 μg/kg BW/day (low dose) or 250 μg/kg BW/day (high dose), or an equivalent volume of sesame oil (control group) on GD10–GD21. Two additional groups of animals were also exposed prenatally to high-dose BPA (250 μg/kg BW) and control treatments to investigate a potential shift in the period of susceptibility for chemically induced mammary cancer at PND100 in the resulting offspring.

Animals were bred, and female rats were observed for the presence of sperm. Once sperm positive (noted as GD0), pregnant females were housed individually in polypropylene cages (BPA-free) with glass water bottles, fed the phytoestrogen-free AIN-93G (growth) diet (Dyets Inc, Bethlehem, PA), and randomly assigned to a treatment group. On the day of birth (designated as PND0), offspring were sexed, and litters were culled to 10 females per lactating dam. The female offspring were weaned on PND21 and continued on AIN-93G diet until PND70, when they were switched to AIN-93M (mature) diet. On PND50 ± 1 and PND100 ± 2, two sets (*n* = 8/treatment group) of identically treated rats were killed in the estrous phase. The fourth abdominal mammary glands were rapidly dissected from live ketamine/xylazine-anesthetized animals prior to euthanasia. One set of mammary glands was frozen in liquid nitrogen and stored at −80°C until assayed via immunoblotting. The contralateral gland was fixed in formalin for paraffin embedding. For each treatment group on both PND50 and PND100, mammary gland samples from individual rats randomly selected from each litter of each treatment group (8–10 rats/treatment group) were used for either immunoblotting or paraffin embedding. Because chemical treatment of a dam during prenatal exposure results in a single exposure group per treatment, one offspring in each litter was treated as a single observation.

### Tumorigenesis experiments

For tumorigenesis experiments, one female offspring from each litter was given a single gavage of 30 mg DMBA/kg BW on PND50 or PND100. This DMBA dose routinely results in a low number of mammary tumors in Sprague-Dawley rats and allows chemicals that predispose for mammary cancer to increase the number of mammary adenocarcinomas (Brown et al. 1998; Jenkins et al. 2009). On PND50, we treated 31 control, 29 low-dose BPA, and 33 high-dose BPA offspring (each from a separate litter) with DBMA; on PND100, 30 control and 28 high-dose BPA offspring were treated with DBMA. Animals were palpated twice weekly to monitor tumor development; data were recorded on palpable tumor latency, location, and multiplicity. Animals underwent necropsy at 12 months of age or when the tumor burden exceeded 10% of BW. All tumors and gross lesions were dissected out and embedded in paraffin for pathological evaluation. Coded slides were classified by a board-certified pathologist (I.A.E.) as to tumor type, tissue of origin, and degree of invasiveness. Histopathologic characterization of mammary neoplastic lesions included carcinoma grade, proliferation index, and malignancy evaluations, as described by Meyer et al. (2005).

### Estrous cyclicity

We monitored estrous cyclicity of female offspring for 22 days, starting at 4 months of age. Vaginal smears obtained daily from lavage fluid (collected by flushing the female’s vagina with phosphate-buffered saline) were examined under a light microscope. The stage of the estrous cycle was determined based on vaginal cytology, as described by Everett (1989). We determined the number of cycles, number of days in estrus, and the cycle length.

### Immunoblotting

Immunoblotting was performed on six to eight biological samples per treatment group, each sample derived from only one rat randomly selected from separate litters per treatment group, as previously described by Rowell et al. (2005), with modifications. Briefly, whole mammary glands were ground in liquid nitrogen and homogenized in RIPA lysis buffer (Pierce Biotechnolgy, Rockford, IL). After homogenization, the samples were centrifuged for 20 min at 16,000 × *g* at 4°C. Equal protein content (40 μg) was loaded onto precast SDS Tris-HCl polyacrylamide gels (Bio-Rad, Hercules, CA). Proteins were wet-transferred to a nitrocellulose membrane overnight. The membrane was then blocked at room temperature, and the primary antibody was added and incubated overnight at 4°C. The secondary antibody and chemilume (Pierce Biotechnology) were added, and protein expression was visualized using film exposures. Densitometry patterns were assessed using Quantity One (Bio-Rad). We used Kaleidoscope Precision Plus Protein and Pre-stained SDS-PAGE Broad Range standards (Bio-Rad) to identify the protein of interest.

### Cell proliferation

Tissue blocks were sectioned at 5 μm onto glass slides. The slides were deparaffinized and rehydrated through a series of xylene and graded alcohol washes. Slides were boiled in citrate buffer for 15 min, incubated in hydrogen peroxide, blocked, and incubated in Ki-67 antibody (Dako, Glostrup, Denmark) overnight in a humidified chamber. After incubating the slides in the secondary antibody, we used the ImmPRESS kit (Vector Laboratories, Burlingame, CA) to determine antigen localization. Positively stained cells were visualized by incubating the slides with 3,3′-diaminobenzidine (DAB) and counterstained with hematoxylin. Slides were dehydrated with graded alcohols, cleared with xylene, and mounted with a glass coverslip. We used six biologically distinct samples derived from individual litters and counted a minimum of three ductal structures per slide. Cell proliferation was identified as intense nuclear staining for Ki-67 protein.

### Apoptosis assay

We determined the rate of apoptosis using the ApopTag Plus Peroxidase *In Situ* Apoptosis Detection kit (Chemicon International, Billerica, MA) according to the manufacturer’s protocol. Cells that stained positive with DAB and exhibited morphological characteristics of apoptosis were counted as positive. We evaluated five biologically distinct samples per treatment and counted a minimum of three terminal ductal structures per slide, for a total of ≥ 3,000 cells counted per treatment group.

### Statistical methods

The time to first tumor (latency) and time to sacrifice (tumor burden ≥ 10% of BW) were analyzed using the LIFETEST and LIFEREG procedures in SAS (SAS Institute Inc., Cary, NC). Survival functions were first estimated for each group using the Kaplan-Meier method and compared across the groups using the Wilcoxon log-rank test and parametrically using survival regression analysis (Collette 2003). Animals that had not developed a tumor by the end of the study or were sacrificed before the end of the study were censored, and the end of study or sacrifice times were treated as censoring times. Tumor multiplicity was analyzed by the Cochran-Armitage trend test.

For cell proliferation and apoptosis, we used the values for stained versus unstained cells to construct a contingency table. Western blots were tested for equality of variance using a two-sample *F*-test, and the appropriate (assuming equal or unequal variance) two-sample *t*-test was used. *p*-Values ≤ 0.05 were considered statistically significant.

## Results

To evaluate dose response, we treated pregnant rats with 0, 25, or 250 μg BPA/kg BW on GD10–GD21. These BPA exposures did not significantly alter body weights of 2-, 7-, 14-, 21-, 35-, 50-, and 100-day-old female offspring (data not shown). Similarly, prenatal exposure to BPA did not significantly alter the time to vaginal opening [32.5 ± 0.3, 34.4 ± 0.3, and 32.6 ± 0.3 days (mean ± SE) for the zero-, low-, and high-dose BPA groups, respectively]. Serum concentrations of 17β-estradiol from 50-day-old female offspring were not significantly different from controls (11.0 ± 1.2, 12.2 ± 2.3, and 14.5 ± 2.5 pg/mL for the zero-, low-, and high-BPA doses, respectively). Likewise, serum progesterone concentrations were not significantly different from controls (11.8 ± 2.1, 11.5 ± 1.2, and 9.4 ± 1.3 ng/mL for the zero-, low-, and high-BPA doses, respectively). Evaluation of estrous cyclicity of adult female offspring did not show significant changes from either BPA dose (data not shown).

### PND50 DMBA treatment and induced mammary carcinogenesis

Using the well-established chemically induced mammary cancer model, we treated female rats with DMBA on day 50, because this age has been shown to be optimum to induce mammary cancer in Sprague-Dawley rats (Russo et al. 1979; Welsch 1985). Palpating for mammary tumors, we found no significant difference for average time to first tumor for rats exposed prenatally to sesame oil only (controls), or to 25 μg or 250 μg BPA/kg BW (mean ± SE, 109 ± 11 days, 116 ± 14, 106 ± 14, respectively). At time of necropsy, we found no significant difference in tumor multiplicity (2.94 ± 0.48, 2.38 ± 0.42, and 2.88 ± 0.40 tumors/rat exposed to 0, 25, and 250 μg BPA/kg BW, respectively) after DMBA treatment at day 50.

### Differential protein expression

**Figure 1:**
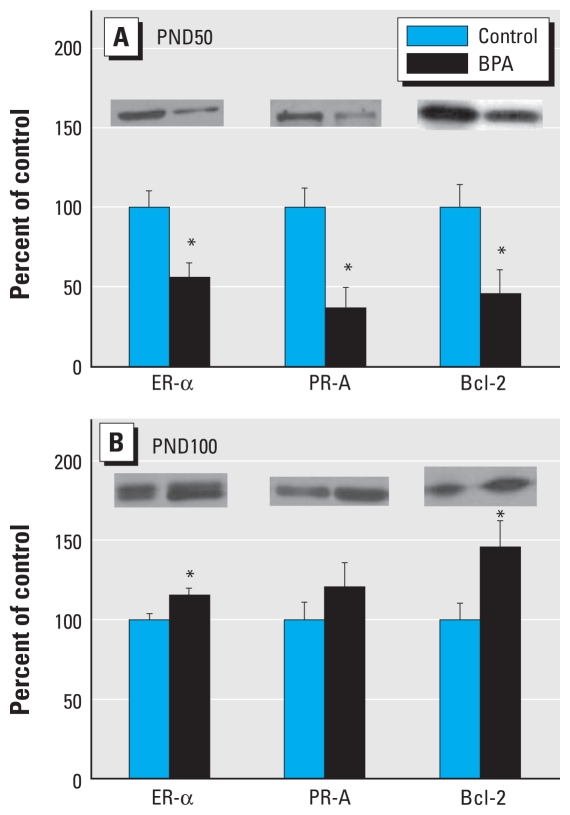
Western blot analysis of ER-α, PR-A, and Bcl-2 in mammary glands of (*A*) 50-day-old and (*B*) 100-day-old rats exposed prenatally to 250 μg BPA/kg BW or an equal volume of sesame oil (controls). Values represent mean density ± SE as a percentage of the control, with densitometry values for controls set to 100; *n* = 6–8 samples per group. Insets are representative immunoblots for each protein per treatment. *p < 0.05 compared with corresponding controls.

We previously reported that the prenatal high BPA dose (250 μg/kg BW) resulted in the maximum number of modulated genes (Moral et al. 2008) and proteins (Betancourt et al. 2010) at day 100 compared with day 50; therefore, we focused our mechanism of action studies on mammary glands of rats exposed prenatally to 0 and 250 μg BPA/kg BW at those two ages. ER-α was significantly down-regulated at PND50 (*p* = 0.002), but up-regulated at PND100 (*p* = 0.042) after prenatal BPA exposure (Figure 1). The expression of two downstream targets of ER (PR and Bcl-2) on both PND50 and PND100 were analyzed as indicators of ER action. On PND50, both PR-A and Bcl-2 were significantly decreased (*p* = 0.003 and *p* = 0.023, respectively) relative to controls. On PND100, Bcl-2 was significantly increased (*p* = 0.01), and PR-A showed a nonsignificant increase (*p* = 0.09) (Figure 1).

**Figure 2:**
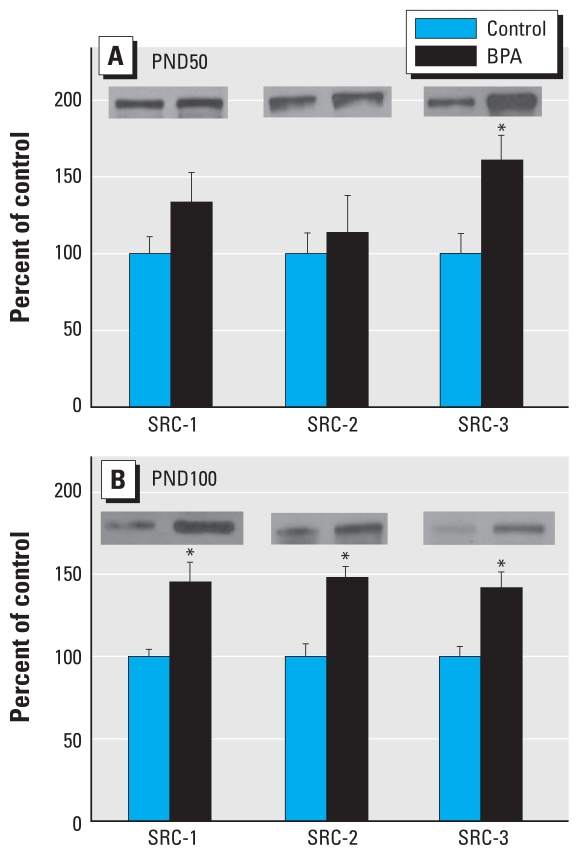
Western blot analysis of SRC-1, SRC-2, and SRC-3 in mammary glands of (*A*) 50-day-old and (*B*) 100-day-old rats exposed prenatally to 250 μg BPA/kg BW or an equal volume of sesame oil (controls). Values represent mean density ± SE as a percentage of the control, with densitometry values for controls set to 100; *n* = 6–8 samples per group. Insets are representative immunoblots for each protein per treatment. *p < 0.05 compared with corresponding controls.

**Figure 3:**
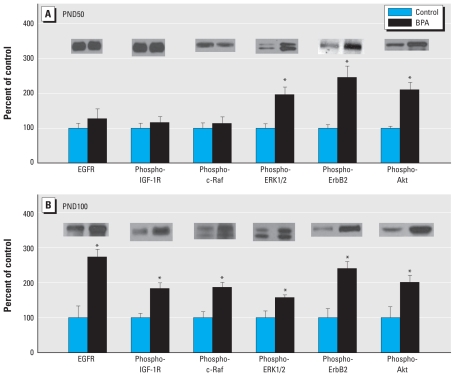
Western blot analysis of EGFR, phospho-IGF-1R, phospho-c-Raf, phospho-ERK 1/2, phospho-ErbB2, and phospho-Akt in mammary glands of (*A*) 50-day-old and (*B*) 100-day-old rats exposed prenatally to 250 μg BPA/kg BW or an equal volume of sesame oil (controls). Values represent mean density ± SE as a percentage of the control, with densitometry values for controls set to 100; *n* = 6–8 samples per group. Insets are representative immunoblots for each protein per treatment. *p < 0.05 compared with corresponding controls.

Because one important factor influencing transcriptional activity of ER-α is the quantity of coregulators present in a given tissue, we determined protein levels of the steroid receptor coactivators SRC-1, -2, and -3 (Figure 2). On PND50, only SRC-3 was significantly increased (*p* = 0.05). However, on PND100, all members of the SRC family up-regulated (SRC-1, *p* = 0.003; SRC-2, *p* = 0.002; and SRC-3, *p* = 0.002). In addition, because prenatal BPA exposure has been shown to increase cell proliferation in the mammary gland of the postnatal rat (Durando et al. 2007), we measured key growth factor receptors and downstream signaling molecules (Figure 3). At PND100, BPA significantly increased expression of EGFR (*p* = 0.0132), phospho-IGF-1R (*p* = 0.007), phospho-c-Raf (*p* = 0.029), phospho-ERKs 1/2 (*p* = 0.030), phospho-ErbB2 (*p* = 0.039), and phospho-Akt (*p* = 0.017). However, on PND50, prenatal BPA exposure significantly up-regulated only phospho-ErbB2 (*p* = 0.018), phospho-ERK 1/2 (*p* = 0.005), and phospho-Akt (*p* = 0.016).

### Cell proliferation and apoptosis

**Figure 4:**
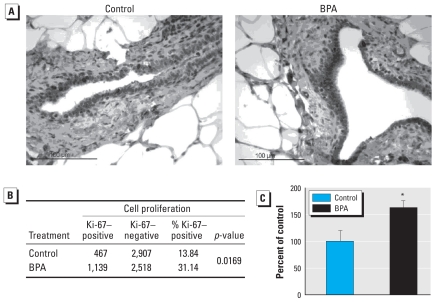
Cell proliferation in mammary glands of 100-day-old rats prenatally exposed to 250 μg BPA/kg BW or an equal volume of sesame oil (controls). (*A*) Ki-67 expression as an indicator of cell proliferation. Ducts from six biologically distinct samples (*n* = 6) were analyzed per treatment; magnification, 40×; bar = 100 μm. (*B*) Contingency table of cell proliferation data. (*C*) Ki-67 labeling index values (mean ± SE) as a percentage of the control group. *p < 0.05 compared with controls.

Because of the significant changes in protein expression on PND100 in offspring with prenatal BPA exposure, we also investigated cell proliferation and apoptosis in mammary glands at PND100. We found increased Ki-67 expression in the epithelial cells, but not the stroma, of mammary tubular ducts of 100-day-old rats prenatally exposed to BPA 250 (*p* < 0.05) (Figure 4) compared with controls. However, prenatal BPA exposure did not alter apoptosis in the mammary glands of these rats (*p* = 0.85) (data not shown).

### PND100 DMBA treatment and induced mammary carcinogenesis

**Figure 5:**
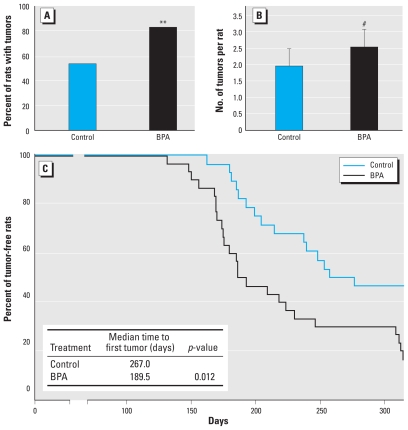
Tumor incidence (proportion of rats that developed at least one tumor; *A*), tumor multiplicity (number of tumors per rat; *B*), and Kaplan-Meier survival curve with median time (days) to first tumor (*C*) in female offspring prenatally exposed to 250 μg BPA/kg BW or an equal volume of sesame oil (controls) and gavaged with a single dose of 30 mg DMBA/kg BW on PND100. Statistical analyses for tumorigenesis are described in “Statistical Methods.” **p = 0.022, and ^#^*p* = 0.070 compared with the control group.

Because prenatal BPA exposure affected more signaling pathways in the mammary glands of PND100 rats than of PND50 rats, our next step was to investigate whether those effects could translate to differences in the susceptibility of the mammary gland to carcinogenesis on PND100 as opposed to PND50. To test this hypothesis, we treated female offspring exposed *in utero* to either sesame oil (control group) or BPA 250 μg/kg BW with DMBA by a single gavage on PND100. Tumor incidence was significantly increased in the BPA group (83.3%) versus the control group (53.6%) (*p* = 0.022), and there was a nonsignificant increase in tumor multiplicity [2.53 ± 0.55 vs. 1.96 ± 0.53 (mean ± SE) in BPA-exposed and control animals, respectively; *p* = 0.07] (Figure 5). Female offspring exposed *in utero* to BPA 250 μg/kg BW also showed significantly decreased tumor latency (*p* = 0.012) compared with the control group (Figure 5C) after DBMA exposure on PND100. In addition, we classified a significantly greater proportion of DMBA-induced mammary tumors as grade II (according to the Bloom-Richardson system) in BPA-exposed animals (9 of 20 tumors; 45%) versus control animals (3 of 13 tumors; 23%) (*p* = 0.0484). A single tumor from each animal was randomly selected for histopathological analysis.

## Discussion

The primary aim of this study was to determine if prenatal BPA exposure would predispose the adult rat mammary gland to carcinogenesis and if the protein signature could provide an insight into the possible targets of BPA in the mammary gland. We choose the DMBA-induced mammary cancer model in Sprague-Dawley rats because there is similarity in ontogeny of mammary gland development and morphology in rats and humans (Russo et al. 1990). DMBA-induced mammary cancer displays pathology similar to human breast cancer, including a common site of origin and a similar pattern of tumor development (McCormick and Moon 1965; Murad and von Haam 1972; Russo et al. 1977), and mammary cancers can be reproducibly induced with a high frequency in this model. In studies of cancer causation or chemoprevention, the standard protocol for administering DMBA is at day 50, because this is within the period (days 40–60) of high mitotic index in the terminal ductal structures of rats (Russo et al. 1983; Welsch 1985). Using this protocol, we found no significant difference in tumor multiplicity or tumor latency in rats exposed prenatally to either of the BPA doses (25 and 250 μg/kg BW) after DMBA exposure on PND50. Based on previous evidence suggesting differences in the mechanism of action of BPA at different ages (Betancourt et al. 2010; Moral et al. 2008), we suspected that the window of susceptibility for DBMA-induced mammary cancer might be shifted from day 50 to day 100 after prenatal BPA exposure. Consequently, we found that DMBA treatment at PND100 resulted in a significantly higher incidence of mammary tumors in rats exposed prenatally to BPA. In addition, relative to control animals, prenatal BPA exposure decreased the latency period (average time to first tumor after DBMA treatment) by 78 days. Furthermore, mammary tumors in the high-dose BPA group (250 μg/kg BW) were more likely than those in control rats to be classified as grade II according to the Bloom-Richardson scoring index, which is based on adenocarcinoma tubular pattern, nuclear grade, and mitotic index (Meyer et al. 2005).

The doses chosen for this study (25 and 250 μg BPA/kg BW to pregnant dams) were based on reported human exposures, experimental studies in rats, and the U.S. Environmental Protection Agency (EPA) maximum acceptable dose. Serum BPA concentrations in pregnant human females have been reported to be 0.46–19 μg/L (Kuroda et al. 2003; Schonfelder et al. 2002; Welshons et al. 2006). Exposure assessments have ranged from 0.2 μg/L (nanograms per gram of tissue) in human fetal cord serum up to 105 μg/L in human placenta (Kuroda et al. 2003). Within the United States, an exposure of up to 50 μg/kg/day (50 ppb) is considered safe by the U.S. EPA, satisfying a 1,000-fold margin of safety (U.S. EPA 1988). Considering metabolism and disposition by the pregnant female rat and dilution to each individual fetus, it is calculated that each offspring would be exposed to 100–1,000 times less BPA than the treated mother. Consequently, the offspring of dams exposed to the high (250 μg/kg BW) and low (25 μg/kg BW) BPA doses would have been exposed to approximately 0.25–2.5 and 0.025–0.25 μg BPA/kg BW/day, respectively (Kurebayashi et al. 2005; Snyder et al. 2000), which is less than the daily tolerable dose of 50 μg/kg BW/day established by the U.S. EPA. In the present study we did not observe any toxic effects on body weight, vaginal opening, estrous cyclicity, and serum estrogen and progesterone concentrations in offspring exposed to either BPA dose.

So why were rats that were prenatally exposed to BPA more susceptible to DMBA carcinogenesis at PND100 than at PND50? Previously, we reported that prenatal treatment with 250 μg BPA/kg BW resulted in a significantly increased number of terminal ducts at PND100 but not at PND50 (Moral et al. 2008). Terminal end buds and terminal ducts are considered the most susceptible terminal ductal structures of the rat mammary gland because of the high mitotic index and undifferentiated state of the cells in these structures (Russo and Russo 1978). Beyond that, terminal ductule hyperplasia is an early lesion present in rat mammary carcinogenesis (Haslam and Bern 1977; Shina and Dao 1975). Our finding of increased Ki-67 expression in the mammary ducts of 100-day-old rats after prenatal exposure to BPA suggests that increased susceptibility to DBMA-induced carcinogenesis is a possible consequence of BPA-mediated effects on cell proliferation. In addition, Markey et al. (2001) reported that mice exposed to 250 μg BPA/kg BW/day by means of Alzet osmotic pumps during gestation had a significant increase in all epithelial structures on PND180. Therefore, greater availability of target structures—in addition to a cellular microenvironment favoring carcinogenesis—could explain the increased tumorigenesis response.

At the molecular level, our previous gene array studies demonstrated that prenatal BPA exposure significantly down-regulated a number of breast differentiation markers at PND100, including *Fabp3*, the homolog to mammary-derived growth inhibitor, a tumor suppressor (Hu et al. 1997; Yang et al. 1994), and whey acidic protein, a mammary differentiation marker (Dandekar et al. 1982). Although it is attractive to explain cancer susceptibility at the gene level, this needs to be validated at the protein level. Using Western blot analysis, we found differential effects of BPA exposure on ER-α and SRC protein expression in the mammary gland. ER-α was down-regulated at PND50, but up-regulated at PND100 in high-dose BPA offspring compared with controls. The expression of downstream targets of ER (PR-A and Bcl-2) (Henriksen et al. 2009) in mammary glands on PND50 and PND100 followed an expression pattern similar to ER-α at each age, suggesting that increased expression of this receptor plays an active role in increased susceptibility to tumorigenic effects of DMBA on PND100. This is also consistent with the fact that DMBA-induced mammary cancer in rats is initially estrogen dependent (Bradley et al. 1976), as we would expect an increase in carcinogenesis with exposure to DBMA at PND100 versus PND50 if there is more ER signaling at that time. Furthermore, we observed that SRCs 1–3 were all up-regulated in BPA-exposed rats compared with controls at PND100, but only SRC-3 was significantly up-regulated at PND50. This shift in the expression of ER-α and the sex steroid coregulators may explain the shift in the window of susceptibility to DBMA-induced carcinogenesis. Our finding of increased steroid receptor coregulator expression after BPA treatment is not without precedent, as we have previously reported that prepubertal exposure to BPA (PND2–PND20) results in up-regulated SRCs 1–3 at 50 days of age (Jenkins et al. 2009). Hence, there is commonality in expression of up-regulated SRCs 1–3 as well as susceptibility for chemically induced mammary cancer, after prenatal and prepubertal BPA exposure.

Our results suggest a plausible mechanism of action by which prenatal BPA exposure could alter signaling molecules, which in turn could increase cell proliferation and contribute to increased susceptibility to carcinogenesis. First, increased levels of EGFR on PND100 suggest the availability of more receptors to activate Ras (Ras-GTP) and subsequently Raf-1, which through a series of intermediate steps results in phosphorylation of the mitogen-activated protein kinases (MAPK) ERK 1/2. These activated kinases then translocate into the nucleus where they phosphorylate specific transcription factors involved in cell proliferation. Second, increased activation of phospho-IGF-1R on PND100 could potentially induce activation of phospho-Akt as well as MAPK signaling pathways that promote cell survival by inhibiting apoptosis. Alternatively, EGFR can induce activation of Akt by forming heterodimers with other members of the ErbB family. Akt activity is elevated in several types of human malignancy, including breast, ovarian, lung, and thyroid cancers (Vivanco and Sawyers 2002). Third, increased activation of phospho-ErbB2 can lead to increased Akt and MAPK signaling. Convergence of the EGFR/erbB2 and IGF-1R signaling pathways could contribute to increased cell proliferation on PND100 and increased susceptibility to tumorigenesis after DBMA exposure on PND100 and explain the lack of BPA effects on tumorigenesis after DBMA exposure on PND50, when only phospho-ErbB2, phospho-Akt, and phospho-ERK 1/2 were increased in BPA-exposed animals. On the other hand, SRCs have been reported to directly influence steroid receptor action, and they have also been suggested as transcription factors that may indirectly promote steroid-mediated gene transcription by increasing growth factor signaling (O’Malley and Kumar 2009). In addition, SRCs have been shown to interact with human epidermal growth factor receptor 2 (HER2/erbB2) (Bouras et al. 2001), and overexpression of HER2 and SRC-3 are both associated with a worse prognosis in cases of human breast cancer (Kirkegaard et al. 2007; McIlroy et al. 2006). We found that prenatal BPA exposure increased the level of ER-α and all three members of the SRC family on PND100 in rats. These effects may translate to increased ER-α signaling and also suggest that cross-talk of SRCs and HER2/erbB2 could further promote an increase in cell proliferation on PND100 that is not present in the mammary gland on PND50 when ER-α is decreased and only SRC-3 is increased. Future research is necessary to confirm this hypothesis.

Additional research is also needed to determine the underlying mechanism linking early exposure to BPA to long-lasting effects on the mature animal. The prevailing hypothesis is that prenatal (or early postnatal) exposure to a hormonally active chemical can permanently alter gene expression by altering DNA methylation or chromatin assembly (Ho et al. 2006; Hsu et al. 2010). Imprinting or organizational effects during early critical periods of development have been described that cause permanent manifestations later in life, even in the absence of the original effector (Baylin et al. 1998; Lamartiniere 2002; Lamartiniere et al. 1982). We believe that imprinting after exposure to BPA and other hormonally active chemicals can determine the biochemical blueprint of mammary tissue responses to future stimuli in adults.
